# Parvovirus B19-Induced Pancytopenia: A Case Report of Aplastic Crisis

**DOI:** 10.7759/cureus.76660

**Published:** 2024-12-31

**Authors:** Francisco de Oliveira Simões, Hugo Goncalves, Rosa Sá, Bárbara Fraga Campos, Rui M Domingues, Narciso Oliveira, Teresa Pimentel

**Affiliations:** 1 Division of Internal Medicine, Unidade Local de Saúde (ULS) Braga, Braga, PRT; 2 Division of Rheumatology, Unidade Local de Saúde (ULS) Braga, Braga, PRT; 3 Division of Oncology, Unidade Local de Saúde (ULS) Braga, Braga, PRT; 4 Division of Internal Medicine, Hospital de Braga, Braga, PRT

**Keywords:** anemia, hematological complications, hypoproliferative anemia, immunocompetent, pancytopenia, parvovirus b19, rash, viral infection

## Abstract

Parvovirus B19 is a common viral pathogen that commonly manifests with mild, flu-like symptoms or an erythematous rash. In rare instances, it may lead to hematological complications, even in immunocompetent individuals.

This report presents a case of a 64-year-old male without prior hematological conditions who presented with generalized malaise, intermittent fever, and a pruritic rash. Initial laboratory findings revealed normochromic, normocytic anemia, leukopenia, thrombocytopenia, and acute kidney injury. Extensive diagnostic workup excluded common bacterial and viral etiologies. Serology for parvovirus B19 demonstrated positive IgG and IgM antibodies, indicative of a recent infection. The patient developed severe pancytopenia during hospitalization. Supportive care was provided, and the patient demonstrated complete clinical and hematologic recovery at discharge..

This case underscores the potential for severe hematological complications of parvovirus B19 infection in immunocompetent individuals. Early recognition of these complications is crucial for optimal management and patient outcomes. Clinicians should include parvovirus B19 in the differential diagnosis of unexplained pancytopenia, even in patients without known hematological disorders or other types of immunodeficiency.

## Introduction

Parvovirus B19 is a non-enveloped, single-stranded DNA virus that typically presents with an erythematous rash, often described as a 'slapped cheek' appearance - its most common presentation, also known as 'erythema infectiosum' - affecting the limbs, trunk, and face while sparing the palms and soles [1,2]. In adults, the rash is less distinctive than in children and can sometimes be mistaken for rubella. Despite this fact, the infection is still most common in childhood. Other common symptoms include headaches, arthralgias, and low-grade fever. In adults, arthralgias generally present as symmetric, usually affecting hands and occasionally ankles, knees, and wrists, and can mimic rheumatoid arthritis. Parvovirus B19 is a virus that induces temporary suppression of erythropoiesis, which is typically mild and asymptomatic, causing transient anemia, and on rare occasions, it may precipitate a transient aplastic crisis.

The authors report a case of a 64-year-old man without any prior hematological history who developed pancytopenia as a complication of a parvovirus B19 infection [3].

## Case presentation

**Figure 1:**
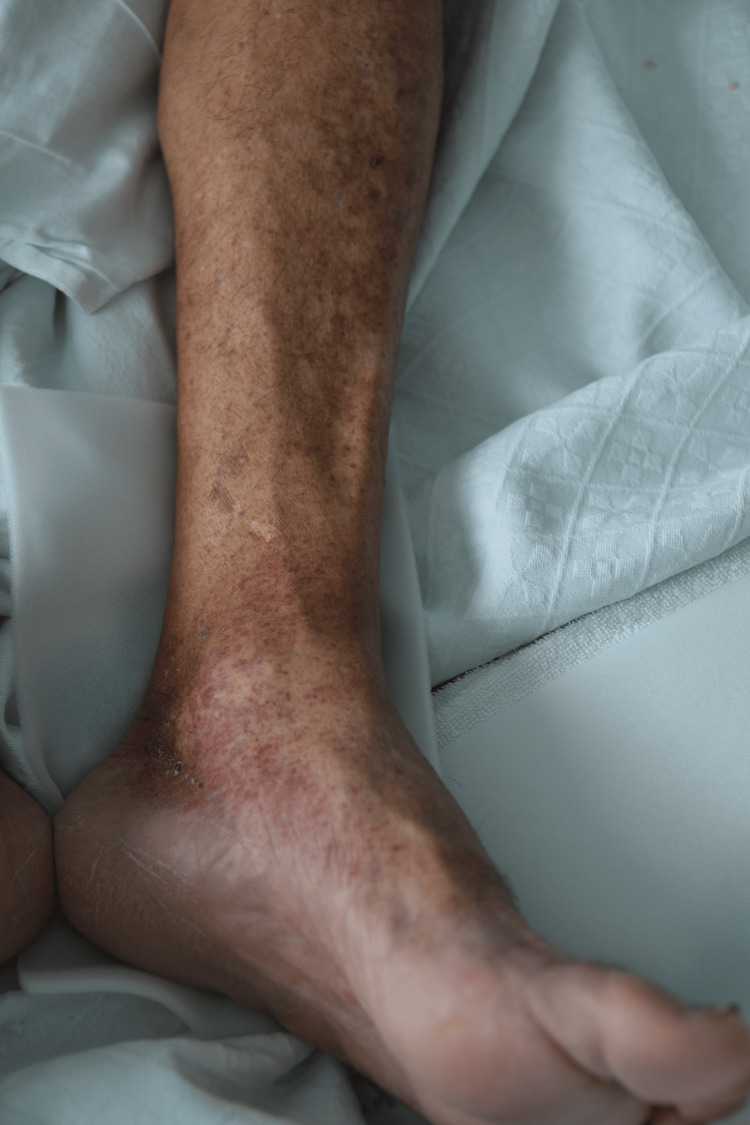
Erythematous rash sparing palms and soles observed in the patient.

**Figure 2:**
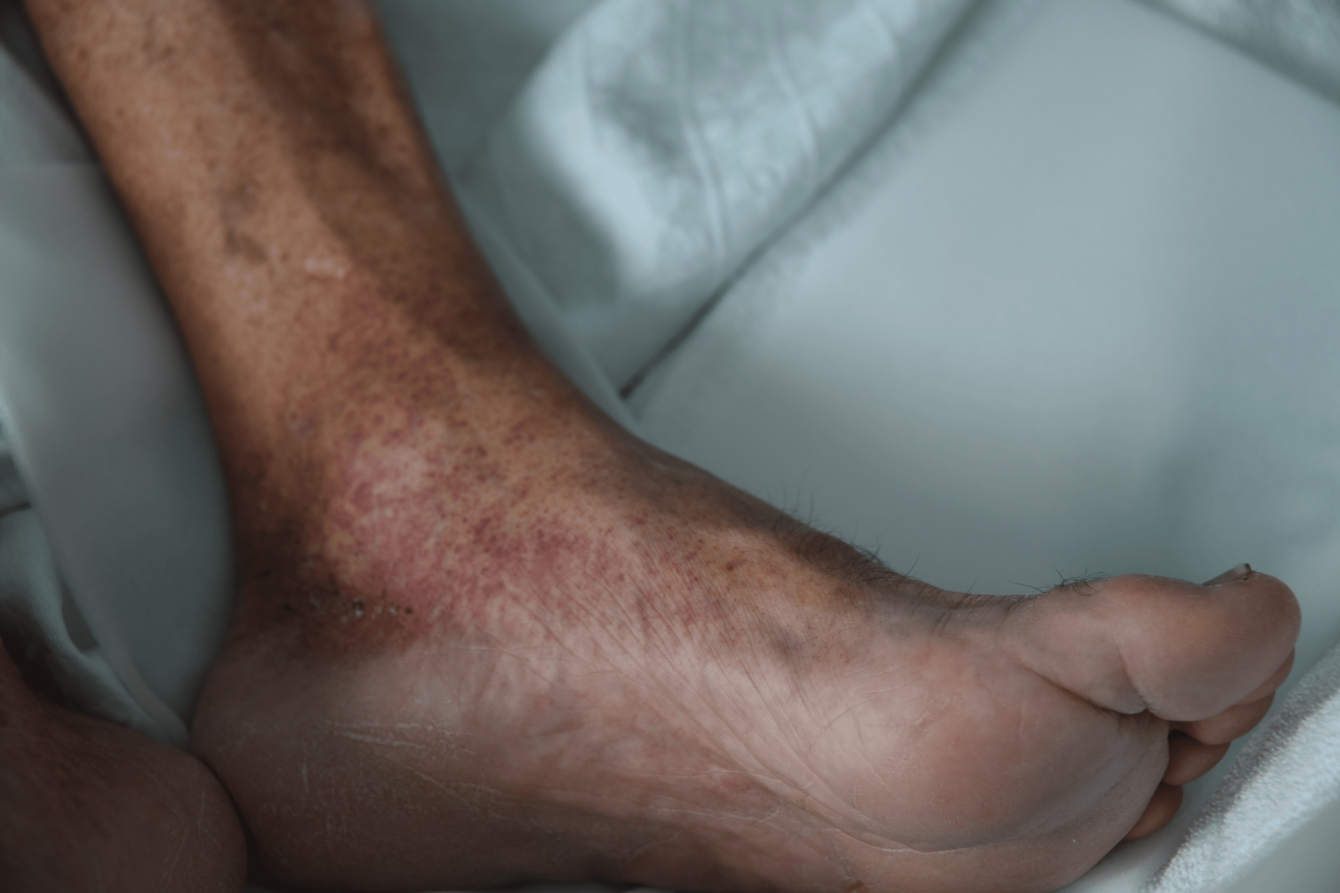
Another angle of the rash presented.

A 64-year-old man with a history of essential hypertension, dyslipidemia, ischemic heart disease, chronic gastritis with gastric metaplasia, nephrolithiasis, chronic degenerative osteoarticular disease, and a history of smoking presented to the Emergency Department (ED). The patient's usual medications included antihypertensives (amlodipine and perindopril), beta-blocker (bisoprolol), statin (atorvastatin), antiplatelet therapy (aspirin), and analgesic (tramadol and paracetamol association). In the ED, he reported generalized malaise, asthenia, myalgias, and diffuse arthralgias that had been evolving over four days. Additionally, he reported intermittent fever episodes occurring every 12 hours, with a peak temperature of 39.1°C, and experienced two episodes of postprandial vomiting. Two days prior to his ED visit, he developed a pruritic rash (Peak Pruritus Numerical Rating Scale of 7/10) initially confined to the medial aspect of the thighs bilaterally, which subsequently spread to the legs, feet, and trunk. He denied experiencing headaches, chest pain, dyspnea, orthopnea, palpitations, cough, or other gastrointestinal disturbances. Upon physical examination, the patient was conscious, alert, and oriented. Vital signs were stable, with normal blood pressure, heart rate, and oxygen saturation of 95% on room air. A punctate erythematous rash was observed, not blanching with pressure, distributed across the body with greater prominence on the limbs. The palms and soles were spared, as shown in Figures 1-2. A cardiopulmonary examination revealed normal heart and lung sounds. Abdominal examination revealed a soft, non-tender abdomen with no organomegaly, masses, or signs of peritoneal irritation.

Laboratory tests performed in the emergency department revealed the following: normochromic, normocytic anemia with a hemoglobin level of 10.5 g/dL, leukopenia with an absolute white blood cell count of 2,300 cells/μL, thrombocytopenia with a platelet count of 40,000/μL, acute kidney injury with a serum creatinine level of 3.6 mg/dL, normal electrolyte levels, and mild liver enzyme elevation with aspartate aminotransferase (AST) and alanine aminotransferase (ALT) levels of 95 and 96 U/L, respectively. The results are presented in Table 1. Arterial blood gas analysis did not reveal any acid-base disturbances. Renal ultrasound did not demonstrate any evidence of renal obstruction, nephrolithiasis, or other abnormalities. Blood cultures, urine cultures, and serological tests for HIV, hepatitis A, B, C, and syphilis were obtained. The patient was subsequently admitted to the Internal Medicine ward for further evaluation.

**Table 1:** Detailed laboratory findings from the patients. Ab: Antibody; ALT: Alanine transaminase; AST: Aspartate transaminase; HAV: Hepatitis A virus; HBV: Hepatitis B virus; HCV: Hepatitis C virus; HIV: Human immunodeficiency virus; HBs: Hepatitis B surface; HBc: Hepatitis B core

Parameters	Laboratory Values at Admission	Laboratory Values at Nadir	Reference Values
Peripheral blood			
Hemoglobin	10.5 g/dL	9.8 g/dL	13.5 - 17 g/dL
Leukocytes	2.3 x10^3^/μL	1.1 x 10^3^/μL	4.0 - 11.0 x10^3^/μL
Platelets	40000 /μL	27000 /μL	150000 - 400000 /μL
Creatinine	3.6 mg/dL		0.70 - 1.20 mg/dL
AST	95 U/L		12 - 40 U/L
ALT	56 U/L		7 - 40 U/L
Ab Anti-HIV I/II	Negative		
Ab Anti-HAV	Negative		
Ab HBs HBV	Negative		
Ab HBc HBV	Negative		
Ab Anti-HCV	Negative		
Syphilis	Negative		
Weil-Felix test	Negative		
Ab Anti-*Leptospira interrogans*	Negative		
Ab Anti-*Coxiella burnetii*	Negative		
Ab Anti-*Rickettsia*	Negative		
Parvovirus B19 IgG	Positive		
Parvovirus B19 IgM	Positive		

During hospitalization, he remained normocardic, normotensive, and afebrile, with an erythematous punctiform rash distributed across the body, sparing the palms and soles, without evidence of inoculation or bite marks. During the hospital stay, the patient experienced worsening pancytopenia, with normochromic, normocytic anemia at 9.8 g/dL, thrombocytopenia with a minimum of 27,000 platelets/μL, leukopenia of 1,100/μL, and neutropenia of 200/μL. Serological tests for HIV, hepatitis A, B, C, and syphilis were negative, as were blood cultures and urinalysis. Given the initial suspicion of a zoonotic infection, serological tests for toxoplasmosis, rickettsioses, leptospirosis, *Leishmania *spp*.*, and *Coxiella burnetii *were also performed and returned negative. The serology for parvovirus B19 showed positive IgG and IgM, indicating a recent infection, leading to the main diagnosis of acute parvovirus B19 infection [4].

These findings confirmed an infectious etiology, with acute parvovirus B19 infection as the most likely diagnosis. During the remainder of his hospitalization, the patient demonstrated progressive clinical and laboratory improvement, with complete resolution of the rash and normalization of all blood cell counts. The patient was discharged from the hospital with a scheduled follow-up in the Internal Medicine clinic. At the follow-up appointment conducted eight weeks later, the patient exhibited a full resolution of all abnormalities, including a complete recovery from pancytopenia.

## Discussion

This case report describes an immunocompetent patient who developed severe pancytopenia as a complication of acute parvovirus B19 infection. Unlike the mild rash observed in children with parvovirus B19 infection, adults, particularly middle-aged women, may develop clinically significant arthropathy [2,3]. Parvovirus B19 typically causes pure red cell aplasia due to its selective destruction of erythroid progenitor cells. The resulting anemia is often mild and self-limiting due to the relatively long lifespan of red blood cells [5]. In patients with underlying hematological disorders, the infection can cause transient aplastic crisis with severe anemia. In this case, the immunocompetent patient without prior hematological conditions showed a marked decrease in all three cell lines, an uncommon and rare finding. The pathophysiology underlying pancytopenia in parvovirus B19 infection is not fully understood [3,5]. Parvovirus B19 exclusively targets human erythroid progenitors, a tropism demonstrated in tissue cultures where the virus selectively inhibits erythroid colony formation without affecting myeloid cells [3]. Potential mechanisms include direct viral infection of other hematopoietic progenitor cells (while primarily targeting erythroid precursors, parvovirus B19 may also infect other hematopoietic stem cells, leading to a decrease in all three blood cell lines) and immune-mediated destruction (the virus may trigger an exaggerated immune response, leading to the destruction of not only infected cells but also other hematopoietic cells) [5]. This case highlights the importance of considering parvovirus B19 infection in the differential diagnosis of pancytopenia, even in immunocompetent individuals without underlying hematological conditions.

## Conclusions

In this case, the immunocompetent patient without prior hematological conditions showed a marked decrease in all three cell lines, an uncommon and rare finding. Thus, this case emphasizes the importance of early detection and appropriate monitoring, which are vital for guiding clinical decisions and improving patient outcomes. Clinicians should maintain a high index of suspicion for parvovirus B19 infection in cases of unexplained cytopenias to ensure timely intervention and prevent potential complications.
